# Muscle Herniation: An Often-Missed Pseudotumor

**DOI:** 10.5334/jbsr.2294

**Published:** 2020-11-13

**Authors:** Jesper Dierickx, Filip Vanhoenacker

**Affiliations:** 1 AZ Sint-Maarten, BE; 2 University (Hospital) Antwerp/Ghent, BE

**Keywords:** muscle herniation, ultrasound, magnetic resonance imaging

## Abstract

**Teaching Point:** Dynamic ultrasound and knowledge of a clinical history of a soft-tissue lump that increases in volume upon muscle contraction or weight-bearing are crucial in the diagnosis of muscle herniation.

## Case Presentation

**Figure 1:**
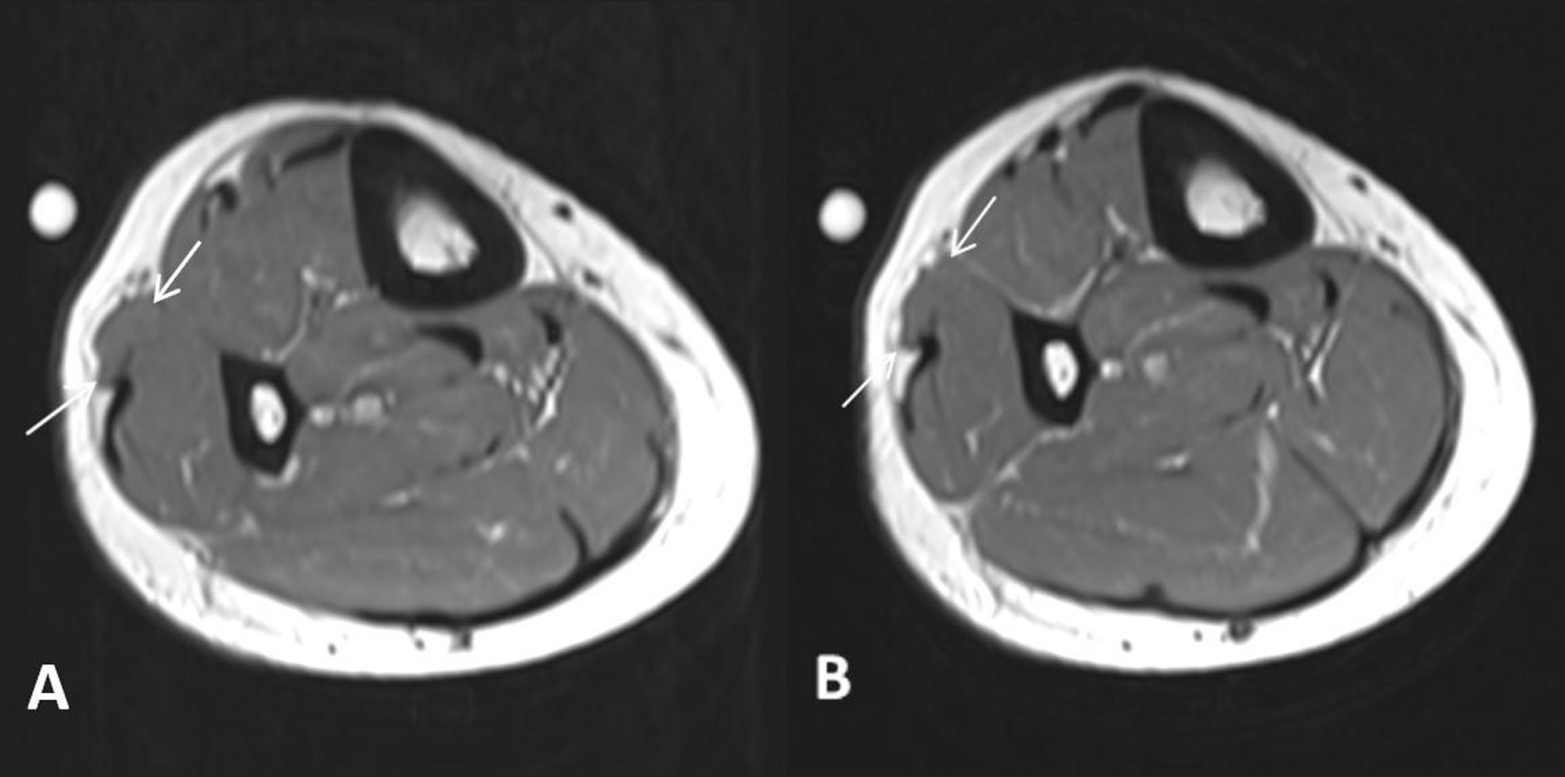


**Figure 2:**
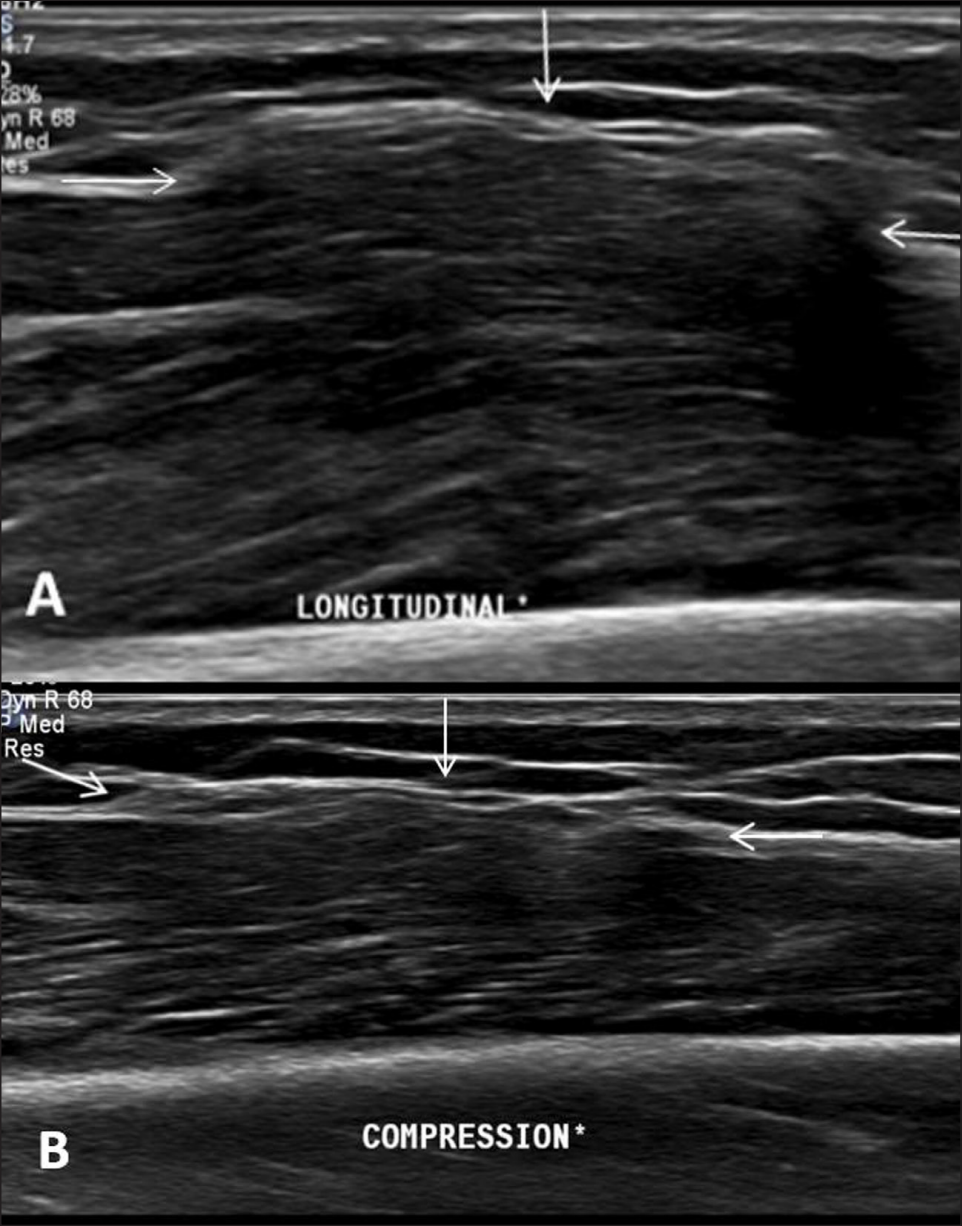


A 42-year-old woman was referred for magnetic resonance imaging (MRI) for evaluation of a painless soft-tissue swelling on the lateral side of the lower leg. The lesion had been present for many years and was more prominent in standing position. There was no history of trauma. A skin marker was placed at the soft-tissue lump in standing position. MRI showed subtle bulging of the contour of the peroneus longus muscle belly (Figure 1A, white arrow). The lesion was iso-intense to muscle tissue on all pulse sequences. There was no volume increase upon peroneus longus muscle contraction in supine position (Figure 1B). Subsequent dynamic ultrasound in standing position confirmed protrusion of peroneus longus muscle tissue (white arrow) through a fascial defect (Figure 2A). The lesion was barely visible in supine position and could be completely reduced upon compression with the ultrasound probe (Figure 2B). The muscle protrusion appeared more pronounced in standing position (supplementary video). Clinical examination and imaging were characteristic of peroneus longus muscle herniation.

## Comment

Muscle herniation consists of a focal muscle protrusion through a myofascial defect [1]. The fascia defect may be either congenital or acquired. The most commonly affected muscles are the tibialis anterior, extensor digitorum longus, peroneus longus and brevis, gastrocnemius, and the forearm flexors [1].

On clinical examination, the lesion usually presents as a painless palpable swelling and is more prominent and harder upon muscle contraction. In some cases, it may be painful during exercise. Dynamic ultrasound is the preferred imaging modality when muscle herniation is suspected. Ultrasound during contraction or in standing position demonstrates volume increase through the myofascial defect compared to ultrasound during rest. Extensive compression with the ultrasound probe may reduce the muscle protrusion. Therefore, a technique of graded ultrasound compression and alternated muscle relaxation is recommended. On dynamic MRI with muscle contraction and relaxation, a bulging muscle contour with a focal fascial disruption may be rarely seen. It is often not recognized due to its supine position. In an uncomplicated muscle herniation, the protruded muscle tissue demonstrates iso-intense signal intensity to non-herniated muscle tissue. Hyperintense signal in the herniated muscle tissue on T2-WI corresponds to muscle edema or fascial tearing [1].

Treatment is not necessary in asymptomatic patients. Activity restriction and support stockings may relieve mild symptoms. Surgical treatment, such as wide fasciotomy, is only required in patients with severe symptoms [1].

In conclusion, radiologists should be aware of the typical clinical history of the patient. For correct diagnosis, a high index of suspicion and dynamic ultrasound using graded compression and contraction are mandatory.

## Additional File

The additional file for this article can be found as follows:

10.5334/jbsr.2294.s1 Supplementary Video. Dynamic ultrasound (longitudinal view) in standing position shows disappearance of the muscle herniation upon compression with the ultrasound probe.
